# Effects of the weight of shoes on calf muscle simulation

**DOI:** 10.1186/s13047-020-00415-x

**Published:** 2020-07-23

**Authors:** I-Lin Wang, Yi-Ming Chen, Ke-Ke Zhang, Ming Gou, Jia-Qi Li, Yu-Hong Jiang

**Affiliations:** 1 College of Physical Education, Hubei Normal University, No. 11, Cihu Road, Huangshi, 435002 Hubei Province China; 2 Graduate College, Jilin Sport University, No. 2476 Ziyou, Changchun, 130022 Jilin Province China; 3 Sports Department, Southwest University of Political Science and Law, No.301 Baosheng, Chongqing, 401120 China; 4 Health Technology College, Jilin Sport University, No. 2476 Ziyou, Changchun, 130022 Jilin Province China

**Keywords:** Running, Individual muscle contribution, Kinematics, Biomechanics, Footwear

## Abstract

The current study investigated the effects of shoes of different weights on calf individual muscle contributions during a running cycle. Twenty male runners ran on a force platform with shoes of four different weights (175 g, 255 g, 335 g, and 415 g). The study evaluated runners’ lower extremity muscle forces under the four shoe weight conditions using a musculoskeletal modeling system. The system generates equality and inequality constraint equations to simulate muscle forces. The individual muscle contributions in each calf were determined using these muscle forces. Data were compared using one-way repeated-measure ANOVA. The results revealed significant differences in the contributions of the gastrocnemius lateralis. Post hoc comparisons revealed that running in the 175 g shoes resulted in a larger contribution of the gastrocnemius lateralis than running in the 415 g shoes during the braking phase. Therefore, wearing lightweight shoes while running may promote fatigue in the gastrocnemius muscle during the braking phase. The calf muscle activation results may indicate that an adaptation period is warranted when changing from heavy to lightweight shoes.

## Background

Shoe weight can be used to categorise running shoes. Shoes with a weight of 150–200 g are called light shoes, shoes with a weight of 200–300 g are called minimalist shoes and shoes with a weight of 360 g or greater are called heavy shoes [1]. Minimalist shoes have been speculated to strengthen foot muscles and arches, which may help prevent injuries [2]. The reasons for this phenomenon may be that impact force on the ground acts as an input signal to trigger muscle tuning [3], and runners use their own comfort mechanisms to maintain their preferred movement path and reduce the risk of injury [4].

Previous studies have noted that reducing the weight of shoes has been found to improve running economy [5]. Previous studies have also observed that adding 100 g of weight per shoe increases the submaximal VO2 (increases oxygen consumption) by ~ 1% [6]. Hoogkamer et al. also found that adding 100 g of mass per shoe increases metabolic rate by 0.75% at a velocity of 3.5 m·s-1 during the 3000 m time-trial running [7]. This concept of compensation for additional shoe weight implies decreased movement efficiency when performing in heavy shoes [8]. Runners with heavy shoes produce more oxygen and consume more energy than those with lightweight shoes, resulting in a low running efficiency [9]. Reducing the weight of shoes increases the efficiency of running because less force must be generated by muscles, and less mechanical work is required. When muscles perform mechanical work, they consume energy. Therefore, shoes of different weights and configurations can change a runner’s foot muscles and energy consumption.

The lack of arch support in minimalist running shoes have been shown to increase the strength of foot muscles [10]. This concept is reflected by an increase in the cross-sectional areas of both intrinsic and extrinsic foot muscles after a period of running in minimalist shoes that mimic barefoot running [11]. Further, use of Vibram FiveFinger minimalist shoes have been shown to increase the intrinsic muscle thickness and strength of the abductor hallucis muscle [12]. These effects have been proposed to reduce the risk of muscle injury.

Different shoes (i.e. different shoe masses) may change a runners foot strike patterns [13], which in turn may affect muscle activation in the lower extremities. Musculoskeletal models may be used as generic models or subject-specific models. The generic models made by generic data measurements from cadaveric quantity information [14, 15]. To date, these effects have not been reported during running with shoes of different weights focus on calf individual muscle contributions.

Therefore, the aim of the current investigation was to examine forces produced by muscles and contributions of calf muscles during the braking phase. The hypotheses were that a large shoe mass would result in increased calf muscle activation during the braking phase of a running cycle. This study may provide important information regarding the extent of the recruitment of key muscles when running with shoes of different weights.

## Methods

### Participants

Twenty males with a mean (SD) age of 21.8 (1.2) years, height of 1.72 (0.03) m and weight of 68.00 (4.32) kg were recruited from a university. The participants had no experience wearing the running shoes used in this experiment (running shoe, model 1208, Xinwei, China). None of the participants reported having any musculoskeletal or ligamentous injuries to the lower extremities at the time of participation or during the 6 months prior to the experiment. Prior to data collection, each participant gave informed consent, as stipulated by the Antai Medical Care Corporation Memorial Hospital (Pingdong, Taiwan; IRB no. 15–066-B1).

### Experimental protocol

**Fig. 1:**
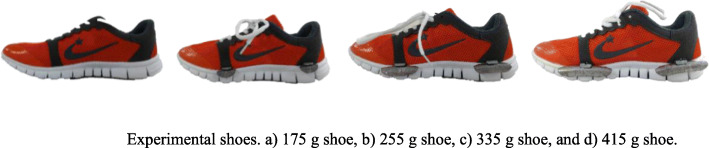
Experimental shoes. **a** 175 g shoe, **b** 255 g shoe, **c** 335 g shoe, and d) 415 g shoe

All participants wore identical running shoes throughout the data collection period to minimize variability. Lead weights were attached to the four outer sides of each shoe to reach the required total weight. Four of the same shoes of different weights were used, and the additional weights were evenly distributed over the length of the shoes in this study (Fig. 1): shoe only (175 g +/− 5 g), shoe alone with an additional 4 × 20 g lead weight (255 +/− 5 g), shoe alone with an additional 4 × 40 g lead weight (335 +/− 5 g), and shoe alone with an additional 4 × 60 g lead weight (415 +/− 5 g). The lead weights were covered with tape, so the participants were blinded to the different footwear conditions. The participants were instructed to warm up for 20 min (by raising their knees high and lowering their jaw) and to practice running five times at a comfortable, controlled, self-selected running velocity with each shoe weight condition for familiarization before data collection [16]. A rest period of 2 min separated the trials. The shoe weight condition order was randomized for each participant.

### Data analysis

**Fig. 2:**
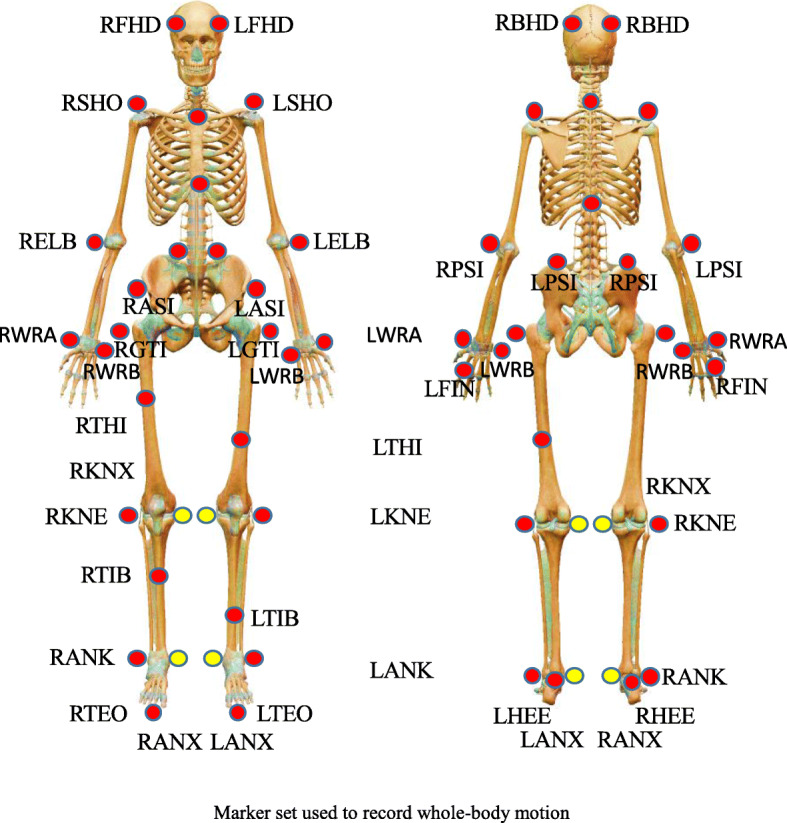
Marker set used to record whole-body motion

A motion capture system (Qualisys Track Manager [QTM], Oqus 100, Sweden) consisting of seven infrared video cameras recorded the marker trajectories at a sampling rate of 200 Hz. Whole-body motion data were collected with a total of 40 reflective markers (19 mm in diameter) placed on bony landmarks (Fig. 2). To form a 10-m-long straight path, two force plates (BP600900, AMTI Inc., Watertown, MA, USA) that measured ground reaction force (GRF) data at a sampling rate of 1000 Hz were embedded in the path. GRF measurements were normalized to the body mass to facilitate interindividual comparisons. The motion and force data were time synchronized with the Qualisys 64-channel interface. The marker trajectories and GRF data were imported into MATLAB (version 7.0; Mathworks Inc., Natick, MA) from QTM software for data reduction and analysis. The kinematic and force data were low-pass filtered with fourth-order Butterworth filters and 12 Hz and 50 Hz cutoff frequencies, respectively. The average data per participant were used for analysis. The dominant leg was defined as the leg used by the participant to kick a ball. The trials were normalized to 100% of the gait cycle on the dominant side for analysis [16]. Muscle simulations are commonly used for the analysis of load during body movements [14, 15, 17]. In this study, the specific muscles used in the comparisons of calf muscle’s contributions during the braking phase among the different shoe weight groups were considered. A musculoskeletal modeling system was adopted to determine muscle forces. Simulations of the muscle forces were generated using the Biomechanics of Bodies (BoB) biomechanical modeling package [18]. The BoB musculoskeletal modeling system was used to generate the equality and inequality constraint equations for a full-body model in a number of arbitrary poses subject to an arbitrary set of external forces. The musculoskeletal model consisted of 508 muscle forces and 30 joint torques [18]. The moments generated by the calf muscle were calculated using inverse dynamics. External forces acting from the ground, gravitational force and inertial forces acting on the participants’ bodies were analyzed and used to calculate the forces in the calf muscle [19]. The individual muscle’s contributions in each calf were determined using these muscle forces. A muscle force optimization approach was utilized in which a solution was sought that minimizes the sum of the square of the activations of the muscles, with activation being defined as the instantaneous force divided by the maximum isometric force of the muscle [20, 21]. The individual calf muscle contributions during the braking phase were calculated using the following formula:

$$ \mathrm{Individual}\kern0.3em \mathrm{muscle}\kern0.3em \mathrm{contributions}\%=\frac{\mathrm{Muscle}\kern0.3em \mathrm{force}}{\mathrm{Total}\kern0.3em \mathrm{muscle}\kern0.3em \mathrm{force}}\ast \% $$

**Fig. 3:**
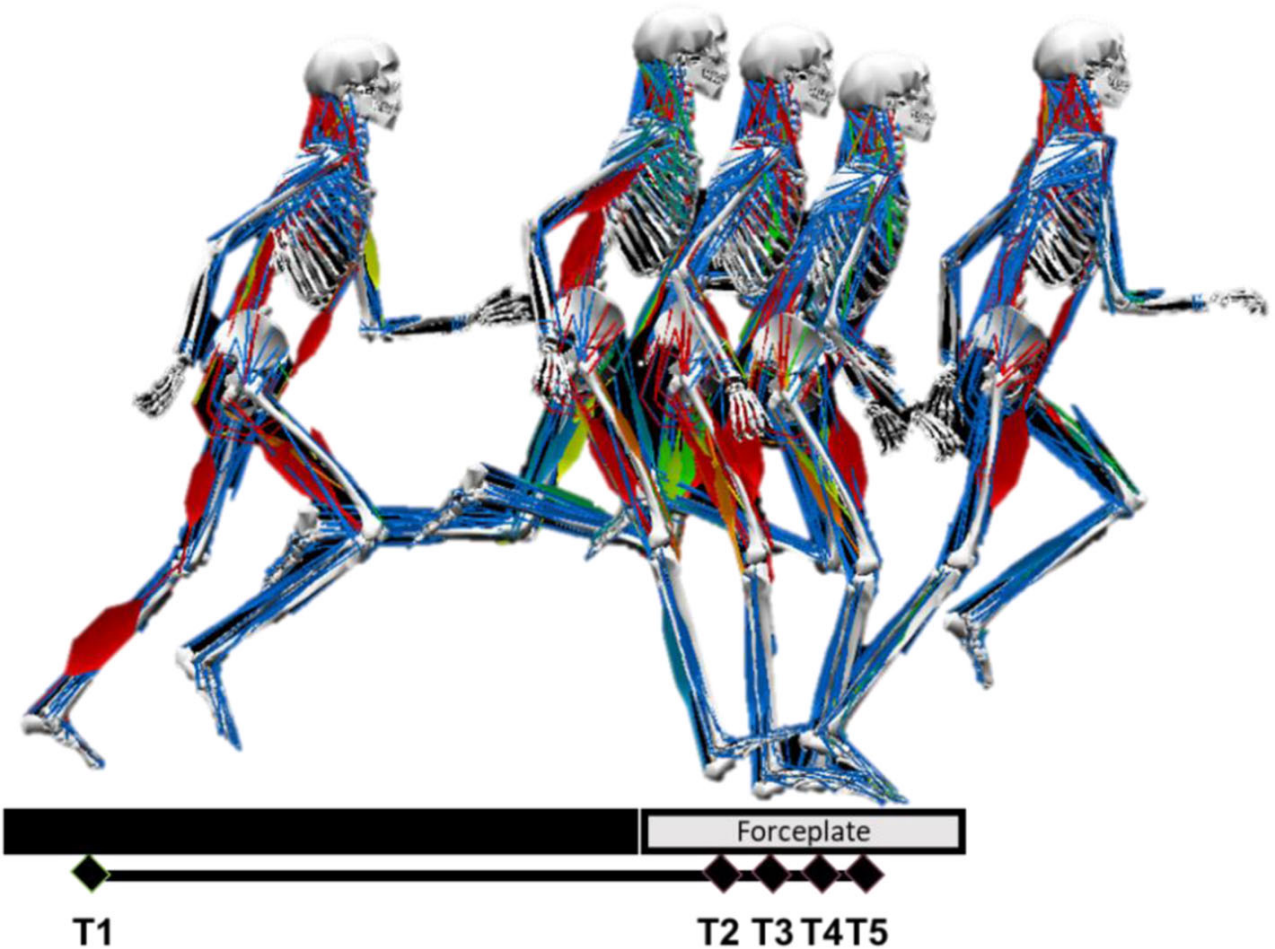
Experimental setup and muscle activations of the lower extremities during the running trials. The following timepoints are represented: (**a**) T1: toe off, (**b**) T2: prelanding, (**c**) T3: contact, (**d**) T4: converse, and (**e**) T5: toe off. The preactivation phase was from T2 to T3, the breaking phase was from T3 to T4, and the push-off phase was from T4 to T5. The stance phase was from T3 to T5

Muscle forces were defined based on the following conditions: (1) the preactivation phase: 50 ms before the foot lands on the force plate; (2) the stance phase: from heel strike (vGRF > 10 Newtons) to when the toe leaves the force plate (vGRF < 10 Newtons); (3) the braking phase: from heel strike to when the anterior-posterior GRF value becomes negative; and (4) the push-off phase: from when the anterior-posterior GRF values are negative and increase to when the foot leaves the force plate (Fig. 3).

### Statistical analysis

The distribution of data were evaluated from skewness statistic for equality of variances. Statistical analysis was performed using SPSS statistics software version 14.0 (SPSS, Inc., Chicago, IL, USA). Repeated-measure one-way ANOVA was used to determine whether muscle forces were higher when participants wore lightweight shoes. The least significant difference (LSD) test was used as a post hoc test [16]. The significance level was set at α = 0.05.

## Results

The results showed that the contribution rate of the lateral gastrocnemius muscle during the braking phase was 5.7% in 175 g shoes, 4.0% in 255 g and 335 g shoes, and 3.1% in 415 g shoes (Table 1). The results indicated that the lateral gastrocnemius contribution of the calf muscle was significantly different during the braking phase.

**Table 1:** Individual calf muscle contributions during the braking phase

Individual muscle contributions modeling during the braking phase (%)	175 g shoes	255 g shoes	335 g shoes	415 g shoes	*p* -value
Tibialis anterior	6.5 ± 0.029	7.2 ± 0.031	7 ± 0.033	6.9 ± 0.029	0.930
Extensor hallucis longus	6.7 ± 0.031	6.9 ± 0.033	6.2 ± 0.034	7 ± 0.029	0.851
Extensor digitorum longus 1st	5.2 ± 0.028	4.6 ± 0.028	6.3 ± 0.024	4.5 ± 0.016	0.195
Extensor digitorum longus 2nd	6.8 ± 0.028	7.2 ± 0.022	7 ± 0.029	5.1 ± 0.023	0.151
Extensor digitorum longus 3rd	6.8 ± 0.030	7.2 ± 0.026	7.2 ± 0.029	6.1 ± 0.026	0.684
Fibularis peroneus brevis	7.4 ± 0.023	6.5 ± 0.026	6.9 ± 0.024	6.5 ± 0.038	0.794
Gastrocnemius lateralis	5.7 ± 0.029	4 ± 0.020	4 ± 0.022	3.1 ± 0.020	0.043*
Gastrocnemius medialis	4.6 ± 0.023	3.7 ± 0.027	3.9 ± 0.016	3.9 ± 0.016	0.717
Soleus	7.8 ± 0.030	6.5 ± 0.034	5.3 ± 0.035	8.1 ± 0.031	0.132
Flexor hallucis longus	6.4 ± 0.022	4.9 ± 0.033	6.8 ± 0.036	6.3 ± 0.028	0.361
Flexor digitorum longus 1st	6.3 ± 0.044	7.4 ± 0.039	6.2 ± 0.021	7.1 ± 0.026	0.792
Flexor digitorum longus 2nd	5.1 ± 0.020	4.2 ± 0.023	5.2 ± 0.025	4.9 ± 0.015	0.516
Flexor digitorum longus 3rd	6.3 ± 0.044	7.4 ± 0.039	6.2 ± 0.021	7.1 ± 0.026	0.792
Tibialis posterior 1st	4.9 ± 0.014	5.5 ± 0.021	5.7 ± 0.022	6.5 ± 0.014	0.194
Tibialis posterior 2nd	4.7 ± 0.009	5.9 ± 0.022	5.3 ± 0.022	5.6 ± 0.016	0.474
Tibialis posterior 3rd	4.7 ± 0.008	5.6 ± 0.019	5.6 ± 0.018	5.6 ± 0.015	0.518
Tibialis posterior 4th	4.1 ± 0.016	5.3 ± 0.026	5.2 ± 0.018	5.3 ± 0.017	0.445

* indicates a significant difference *p* < 0.05

The lateral gastrocnemius muscle contributions results showed significant differences between the shoe weight groups (*p* = 0.043). The post hoc comparisons revealed that the lateral force contributions of the gastrocnemius muscle during the braking phase was larger in the 175 g shoe condition than in the 415 g shoe condition (*p* = 0.023). There were no other significant differences in other muscle contributions throughout the gait cycle.

## Discussion

This study assessed the differences in the forces produced by skeletal muscles and activation patterns of calf muscles during the braking phase while participants ran wearing shoes of different weights. A previous study showed that compared to running while wearing shoes, running barefoot results in higher activation of the gastrocnemius muscle [22]. Similarly, another study found that in the time of peak activity of the lateral gastrocnemius, running with heavy shoes significantly delayed the gait cycle by approximately 4% [23]. However, J Becker, BJJoE Borgia and Kinesiology [24] found no difference in lateral gastrocnemius muscle activation during the whole gait cycle. In this study, differences were found in the braking phase, which may indicate that different shoe weights can change muscle activation during the braking phase. The inter-joint coordination risk of running injuries usually occurs in the braking phase [16]. The increasing muscle activity leads to an increasing load on that muscle [22], and an increasing load might cause injury to the Achilles tendon, which is highly vulnerable to repetitive overload during running activities [25, 26]. Therefore, wearing a light shoe that results in the excessive activation of the gastrocnemius lateralis during the braking phase may increase the incidence of muscle injuries.

Minimalist running shoes can influence the role each muscle has in controlling the motion of the body, with a trend towards higher muscle forces in the gastrocnemius and soleus muscles and higher energy transfers [27]. An appropriate shoe affects the landing impact force when running, which can trigger muscle tuning [3] to allow the skeleton to move in its preferred path through self-determined comfort mechanisms [4]. Minimalist shoes are lighter than traditional shoes, so when people run in minimalist shoes, the gastrocnemius lateralis activates more during the braking phase. The lightest shoes are designed to mimic barefoot conditions. A previous study compared muscle simulations of running in shoes and running barefoot and found that the peak muscle forces of the vastus medialis, vastus lateralis, rectus femoris, and tibialis anterior were larger in the barefoot condition. When barefoot running was simulated, the gastrocnemius had a large peak muscle force [13]. In this study, shoes with the same structure and different weights were compared, and the gastrocnemius lateralis was found to have the largest muscle contributions. Therefore, a light shoe may also cause fatigue in the gastrocnemius lateralis.

Minimalist and lightweight shoes are associated with decreased knee extensor individual muscle contributions and increased ankle joint angles [28]. These factors increase the load on the ankle joint and increase the contribution of the triceps surae muscles [28]. The 175 g shoes may cause greater activation of the gastrocnemius lateralis. A previous study showed that running extreme distances requires more training of the gastrocnemius muscles to prevent muscle fatigue and reduce the risk of injury [29]. The results of this study show that the lightest shoes resulted in the largest muscle contributions, so wearing lightweight shoes likely results in more gastrocnemius fatigue compared to wearing heavy shoes. Participants showed more FFS when wearing the 175 g shoes than when wearing the 415 g shoes [30]. Medial and lateral gastrocnemius activity increases during FFS running, and these heightened activity levels may lead injury because of the overuse of the medial and lateral gastrocnemius muscle [31]. Therefore, wearing lightweight shoes without training may increase the likelihood of injury. This is consistent with previous research which found that transitioning form a RFS to a non-rearfoot strike will reduce running economy and increase loads at the ankle and ankle plantarflexors [32]. Therefore, as wearing lightweight shoes may change a runners strike pattern, it may also increase the risk of injury at these sites. Immediate changes lightweight shoes may cause muscle damage due to the excessive fatigue of the ankle joint muscles during the initial wear period. There are limitations to this study, including the use of simulated muscle models rather than EMG data. Furthermore, an attempt was made to blind the participants, as stated in the methods; however, the runners were clearly not blinded to the weights of the shoes since they could see the weights attached. This factor could have introduced performance bias.

## Conclusion

This study explored the differences between running shoes with the same structure but different weights. The contribution of the gastrocnemius lateralis, i.e., the integral of the force generated, increased for lightweight shoes. Therefore, wearing lightweight shoes may promote muscle fatigue in the gastrocnemius during the braking phase. Calf muscle activation may be indicative that an adaptation period is warranted when changing from heavy to lightweight shoes.

## Data Availability

The datasets used and/or analyzed during the current study are available from the corresponding author upon reasonable request.

## Abbreviations

RFS: rearfoot strike
MFS: midfoot strike
FFS: forefoot strike
GRF: ground reaction force
COM: center of mass
